# Immunogenicity of inactivated *Escherichia coli *O157:H7 with Stx2B microparticle in mice 

**DOI:** 10.22038/IJBMS.2022.63775.14053

**Published:** 2022-09

**Authors:** Nasim Arshadi, Seyed Latif Mousavi Gargari, Jafar Amani, Shahram Nazarian

**Affiliations:** 1 Department of Biology, Faculty of Basic Sciences, Shahed University, Tehran, Iran; 2 Applied Microbiology Research Center, Systems Biology and Poisonings Institute, Baqiyatallah University of Medical Sciences, Tehran, Iran; 3 Department of Biology, Imam Hussein University, Tehran, Iran

**Keywords:** Chitosan, Escherichia coli O157:H7, Inactivated vaccine, Oral vaccine, Shiga toxin

## Abstract

**Objective(s):**

Vaccination using inactivated bacteria is one of the most effective ways to protect against EHEC infection. *Escherichia coli* O157:H7 infections are mainly influenced by Shiga toxins (Stx) and attaching/effacing factors. Among various factors, Stx2B is gaining much attention as a vaccine candidate. Formulating an inactivated bacteria with a suitable adjuvant increases vaccine efficacy and antibody production and can lead to a lasting immune response and protection against O157:H7.

**Materials and Methods:**

To assess vaccine efficacy, in this study, we have considered heat and formalin-inactivated bacteria along with chitosan-coated Stx2B/ Stx2B in a mouse model. Ionotropic gelation via tripolyphosphate anions was used to coat Stx2B on chitosan. Subcutaneous injection or oral gavage was used to immunize mice, which were then challenged with *E. coli* O157:H7.

**Results:**

Immunity and protection against *E. coli* O157:H7 were achieved by all forms of the vaccine. Inactivated *E. coli* O157:H7 formulated with chitosan-coated Stx2B effectively evoked humoral and mucosal immune responses. However, minimum shedding appeared with the mice groups orally immunized with formalin-inactivated bacteria sublimated with chitosan-coated Stx2B and heat-inactivated bacteria plus Stx2B in subcutaneous immunization.

**Conclusion:**

Administration of inactivated whole-cell and toxin was synergistic and increased the protection capacity with both parenteral and oral immunization routes.

## Introduction

Food-borne Enterohemorrhagic *Escherichia coli* (EHEC) infections are a major public health concern (1). Among different serotypes of EHEC,* E. coli *O157:H7, a zoonotic enteric pathogen, causes sporadic infections and outbreaks (2).

 Of various *E. coli *strains*, *the O157:H7 strain is responsible for the majority of infections in humans. In many developing countries such as Argentina (3), diseases caused by *E. coli *O157:H7 may cause chronic renal failure in children and life-threatening hemolytic uremic syndrome (HUS) caused by Shiga toxins (Stx). Diarrhea and hemorrhagic colitis are also among the well-known symptoms of infection.

Furthermore, in developed countries such as the U.S., *E. coli* O157:H7 was responsible for 390 outbreaks between 2003 and 2012, causing 4,928 infections, 1,272 hospitalizations, and 33 mortalities as reported by the Centers for Disease Control and Prevention (CDC) (4).

Fecal-oral transmission by direct contact with human or animal feces or indirect contact with contaminated food, water, or soil is the most common route of *E. coli* O157:H7 infection. However, contaminated food is the primary source of infection (5). 

The main reservoir of EHEC is ruminants, primarily cattle, and humans are infected through direct or indirect contact with their feces (6). Thus, vaccination of ruminants or improved breeding practices to eliminate EHEC from the gastrointestinal tract of the animals has been the main approach to countering this disease (7).

Stx and the formation of attaching and effacing (AE) lesions on enterocytes are among EHEC’s most essential virulence strategies (8). Stxs are among the AB_5_ family of cytotoxins. The toxin’s structure includes polypeptide A, which has enzymatic activity and five copies of B or cell-binding polypeptide. The toxin induces cytotoxicity by inhibiting protein synthesis through N-glycosidases action and depurination of ribosomal RNAs responsible for protein elongation (9). One or two types of Stx toxins are produced by *E. coli *O157:H7, which are classified into separate groups, namely Stx1 and Stx2 (10). Although either toxin can cause human disease, Shiga toxin-producing *E. coli* (STEC), which produces Stx2 or a variant of Stx2, is the leading cause of infection in the U. S. (9).

Antibiotic therapy against STEC may induce the phage lytic cycle; for example, mitomycin C interferes with DNA replication which induces an SOS response that leads to prophage induction (11). Moreover, experiments demonstrated increased Stx production after treatment with antibiotics such as mitomycin C and quinolones. Hence, other approaches, such as attenuated or inactivated bacterial vaccines, are being considered to battle EHEC infection (12).

Heat or chemicals like formalin are commonly used techniques for producing whole-cell inactivated bacterial vaccines (13).

Delivery of the candidate vaccine to the appropriate site can significantly impact the vaccine’s efficacy. Micro and nanoparticles are among the most successful mucosal delivery systems for vaccines. So far, various nanoparticle platforms have been introduced, with chitosan being one of the most widely used vaccine adjuvants and delivery systems. This broad application of chitosan can be attributed to its unique properties, such as low toxicity and biodegradability. Moreover, chitosan has immunostimulatory and high mucoadhesive properties, making it an ideal candidate for delivering gastrointestinal vaccines (14).

Previous reports demonstrated protective immunity in mice after administration of chitosan nano- or micro-particles loaded with plasmid DNA (15). Chitosan nanoparticle alternatives such as chitosan/tripolyphosphate (CS/TPP) have also been used as a delivery system for peptides and proteins (16). For example, Marcili *et al.* (17) have used the ionic gelation process to polymerize Stx2B using sodium tripolyphosphate as a negatively charged molecule and CS as polycation. Here, we have assessed the efficacy of formalin and heat-inactivated bacteria combined with polymerized Stx2B in inducing protective immunity in mice against colonization of *E. coli * O157:H7.

## Materials and Methods

***Plasmid, bacterial strains, and culture conditions***

*E. coli* O157:H7 ATCC: 35218 and plasmids were stored at −80 °C in a freezing media comprising Luria-Bertani(LB) broth and 20% glycerol.

*E. coli* transformants were grown in LB medium containing 70 µg/ml kanamycin.

Methods described by Sambrook *et al*. (18) were used for DNA extraction, transformation, and other molecular techniques used in this study 

***Strain characterization***

The rfbE gene was amplified from the extracted genomic DNA of *E. coli *O157:H7 and used for strain conformation.

The Stx2B clone was kindly gifted by Kazemi *et al*. (19). The clone (Pet28a-Stx2B) was confirmed by PCR before expression. Table 1 contains the sequence of primers used for strain confirmation and Stx2B cloning in this study.

***Preparation of formalin and heat-inactivated bacteria***

Formalin-killed *E. coli *O157:H7 were prepared as described previously (19). 

Bacteria were cultured in LB and harvested with sterile phosphate buffer saline (PBS, pH 7.2) by centrifuging at 8000 × g for 15 min at 4 °C.

The bacterial suspension was inactivated with 0.4% formalin at 37 °C for 1 hr in the shaker and then incubated for 18 hr at 4 °C. The formalin-killed pathogen was centrifuged at 10000 × g for 30 min at 4 °C. After thrice washes by PBS, the concentration of bacteria was adjusted to 1.0 × 10^9 ^CFU ml^−1^ and 1.0 × 10^8 ^CFU ml^−1^ and stored at −20 °C for further use. 

Heat-killed *E. coli *O157:H7 were prepared as described previously (19). 

Briefly,* E. coli *O157:H7 was resuspended in PBS and centrifuged at 7,000 g for 15 min. The obtained pellet was washed three times using PBS and adjusted to 1.0 × 10^9 ^CFU ml^−1^ and 1.0 × 10^8 ^CFU ml^−1^. To prepare heat-killed *E. coli *O157:H7, bacteria were incubated at 70 °C for two hours at standard pressure.

***Expression, purification, and evaluation of Stx2B***

Transformed bacteria harboring pET28a-stx2B were grown in LB broth containing 70 µg/ml kanamycin. When bacteria reached the log growth phase, IPTG (1 mM, Sigma) was used to induce protein expression for 6 hr at 37 °C. Cells were then harvested by centrifugation at 5000×g /15 min, resuspended in lysis buffer (100 mM NaH_2_PO_4_, Tris 10 mM, Urea 8M, pH 8.0), and lysed by sonication on ice. Cell debris was then removed by centrifugation at 13,000×g for 20 min. The expression of recombinant Stx2B was analyzed by electrophoresis on a 15% SDS-PAGE and purified using Ni-NTA (Qiagen) agarose. Bradford technique was used to estimate protein concentration. The total protein from the SDS-PAGE gel was transferred onto a nitrocellulose membrane (Millipore Co, Bedford, MA, USA) for western blot analysis. Anti-6His tag monoclonal antibody (1:10000 dilution, Abcam) was used to detect the recombinant protein on the membranes. Diaminobenzidine tetrahydrochloride (DAB) (Sigma) was used as a chromogen to develop the immunoreactive signal.

***Preparation of microparticle***

Chitosan microparticles were synthesized via ionotropic gelation of chitosan with TPP anions. TPP solution was prepared at a 2.0 mg/ml concentration with deionized water. Chitosan was dissolved in 2% acetic acid solution at a 5 mg/ml concentration and sonicated until the solution became transparent. To start the reaction, 600 µl of Stx2B was added dropwise to 600 µl of the chitosan solution under magnetic stirring (1000 × g, 20 min) at 4 °C. Chitosan microparticles were spontaneously fabricated with the dropwise addition of 500 µl of the TPP to chitosan- Stx2B under magnetic stirring (1000 × g, 1 hr) at 4 °C. Microparticles were separated by centrifugation at 11,000 × g for 20 min at 4 °C. The supernatant was discarded, and the wet pellet of CS/TPP microparticles was collected (16, 20, 21).

***Particle size and morphology***

 Colloidal systems stability is often estimated using Zeta potential measurements. 

Average size and polydispersity index were measured by dynamic light scattering (DLS), estimated from the autocorrelation functions (not shown) using the cumulant analysis. Narrow size distribution is characterized by a low polydispersity index value. Malvern Zetasizer Nano ZS was employed to assess size and potential. Samples were diluted appropriately in disposable polystyrene cuvettes and used for scattering intensity measurement at 25 °C (22).

***Encapsulation rate***

Centrifugation (11,000×g for 20 min at 4 °C) was used to isolate chitosan–TPP microparticles loaded with Stx2B from the non-encapsulated particles. Stx2B concentration was measured using Bradford protein assay. The equation: EE%=Total amount of Stx2B – the free amount of Stx2B/Total amount of Stx2B was used to calculate encapsulation efficiency (EE) using the total amount of Stx2B added in the microparticle preparation and the amount of unloaded Stx2B in the supernatant (23).

***In vitro Stx2B release***

After removing the supernatant, Stx2B loaded chitosan microparticles were collected and resuspended in 1 ml PBS (pH 7.2). The particle suspension was put in an orbital shaker (FINEPCR) under continuous shaking at 25 °C. At predetermined time intervals (1, 2, 4, 6, 10, 15, 48, and 72 hr), each sample was centrifuged, and 100 µl of the supernatant was removed and replaced with 100 µl fresh PBS solution. The concentration of the released Stx2B in the supernatant was determined using the Bradford protein assay. All measurements were performed in triplicate (24).

***Animals***

Five seven-week old female BALB/c mice used in this study were obtained from the Razi institute (Karaj, Iran). Mice were housed with food and water *ad libitum* and under caring staff following the standard rules of the National Institutes of Health guide for the care and use of laboratory animals (NIH Publications No. 8023, revised 1978).

The efficacy of prepared vaccines was assessed by measuring the rate of bacterial shedding after challenging orally, or subcutaneously vaccinated mice (n=40) with killed *E. coli O157* plus Stx2B/ chitosan coated Stx2B.

***Vaccination***

Female BALB/c mice weighed 20–25 g and were divided randomly into two groups for different experiments. For the first experiment, twenty mice were randomly allocated into four groups, All antigens (bacterial suspensions and\or proteins) were resuspended in PBS and emulsified with an equal volume of Alum adjuvant (Razi institute, Karaj) for subcutaneous immunization. The final preparation for each subcutaneous injection contained 1.0 × 10^8 ^(CFU\dose) and 20 µg protein).

Subcutaneous immunizations were given four times with two-week intervals.

The second experiment used a similar design; the only difference was the use of oral immunization and the addition of chitosan-coated Stx2B. 

In the second experiment, twenty mice were divided randomly into four groups; before oral administration, 100 µl of sodium bicarbonate (10%) was gavaged to neutralize the acidic content within the mouse stomach. Each oral vaccine included 1.0 × 10^9 ^bacteria (CFU\dose) and 70 µg protein.

Oral immunizations were given four times, one week apart.

After 10 days, all mice groups were challenged with 10^10^ CFU of living *E. coli *O157:H7 by oral route, and mice groups were monitored daily for 21 days post-challenge. Immunization of different mice groups is exhibited in Table 2. 

***Sample collection***

Blood samples were collected by retro-orbital plexus bleeding, and sera were isolated by centrifugation at 5000 × g for 15 min at 4 °C.

Total IgA levels were assessed using Fresh fecal pellets. For every 100 mg wet weight of fecal pellets, 1 ml of extraction buffer (ice-cold PBS pH;7.2, 0.5% Tween, and 0.05% sodium azide) was used**. **The prepared solution was emulsified for 15 min at room temperature. Debris was removed by centrifugation at 3000 × g for 15 min at 4 °C. The supernatant was isolated and mixed by vortexing with glycerol and phenylmethyl sulphonyl fluoride (PMSF, Sigma) solution. After centrifugation, supernatants were isolated under sterile conditions and stored at -20 °C.

***Estimation of antibody titers by enzyme-linked immunosorbent assay (ELISA)***

Direct and indirect ELISA was used to assess serum IgG, IgA, and mucosal IgA.

For direct ELISA, Polystyrene 96-well plates (Jet biofilm) were coated overnight at 4 °C with 5 µg of Stx2B protein in coating buffer (64 mM Na_2_CO_3_, 136 mM NaHCO_3_, pH 9.6). Wells were washed 3 times with PBST (PBS containing 0.05% Tween 20).

Each well was coated with 10^8^ CFU of an appropriate bacteria for indirect ELISA in 100 µl of the coating buffer at pH 9.6 and incubated overnight at 4 °C. Wells were dried using a hair dryer and washed three times with PBST. Wells were then blocked at 37 °C for 1 hr using 100 µl of 1%BSA in PBST. Various negative controls were included by serially omitting key ELISA components. Serum samples and fecal pellet extracts were serially diluted (1:100 to 1:25,600, or1:1 to 1:128, respectively) and incubated for 2 hr at 37 °C. All wells were then washed, and appropriate goat anti-mouse IgG (Razi, 1:15000) or IgA (sigma, 1:1200) conjugated with horseradish peroxidase were added. Incubation was done for 1.5 hr/37 °C, followed by adding freshly prepared TMB substrate solution (Mono bind) to each well. Incubation was done in the dark for 10 min at room temperature. The reaction was stopped by the addition of 3 N sulfuric acid.

ELISA reader (Sunrise remote, Tecan-Austria) was used to measure absorbance at 450 nm. All samples were run in triplicate.

***Challenging the immunized mice***

Ten days after the last immunization, mice were given drinking water containing streptomycin sulfate (5 g/L) for 24 hr and then fasted overnight. Mice were infected orally with 10^10^ CFU of *E. coli* O157:H7. Fecal pellets were collected at two-day intervals for three weeks. Serial dilutions of the fecal pellet (1ml of LB broth per 0.1 g of fecal matter ) were prepared in PBS. The viable *E. coli* O157:H7 were enumerated by plating onto sorbitol MacConkey agar plates containing tellurite. PCR amplification of the rfbE gene was used to detect the presence of O157 antigen in bacterial colonies (19).

***Statistical analysis***

GraphPad Prism version 6.01 (GraphPad Software Inc., California, USA) was used for data analysis. All values were log10 transformed, and geometric means were used for the analysis. Mean values multigroup comparisons were performed using two way ANOVA test. All experiments’ *P*-values of less than 0.05 were considered statistically significant.

## Results

***Strain characterization***

This study used biochemical tests and PCR to confirm each strain. The rfbE gene is specific to the *E. coli* O157 serogroup.

***Stx2B protein expression and purification***

**Figure 1:**
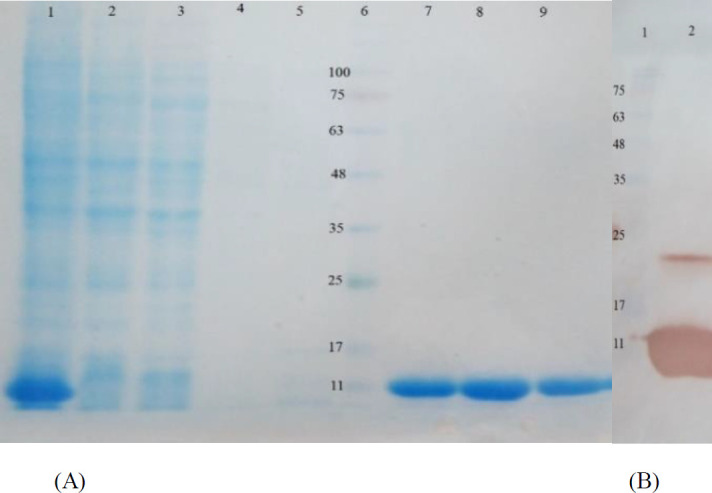
Expression, purification and Western blot analysis of recombinant Stx2B, (A) Purification of recombinant Stx2B protein with 6X-His-tagg by affinity chromatography using Ni-NTA column. Lane 1, Clear cell lysate. Lane 2, flow-through. Lane 3-5, column washed with 40, 50 and 60 mM imidazole respectively, Lane 6, protein weight marker. Lanes 7-9, purified protein after elution with 250 mM imidazole, (B) Western blot analysis of recombinant Stx2B (rStx2B) using anti 6X-His-tag antibodies. Lane 1, protein weight marker. Lane 2, rStx2B

Expression of the Stx2B was analyzed in culture supernatants and cell lysates from *E. coli* BL21 (DE3) cells by SDS-PAGE. Recombinant Stx2B was purified under denaturing conditions, and SDS-PAGE analysis showed a significant band at 11 KD in all the eluted fractions, as shown in Figure 1A. The protein concentration was 0.7 mg/ml. Western blot analysis using anti-His-tag antibodies confirmed the expression of recombinant Stx2B protein (Figure 1B).

***Size and zeta potential of chitosan microparticles and Stx2B-loaded microparticles***

Chitosan microparticles were prepared using ionic gelation interaction between positively charged chitosan and negatively charged TPP at 4 °C.

**Figure 2:**
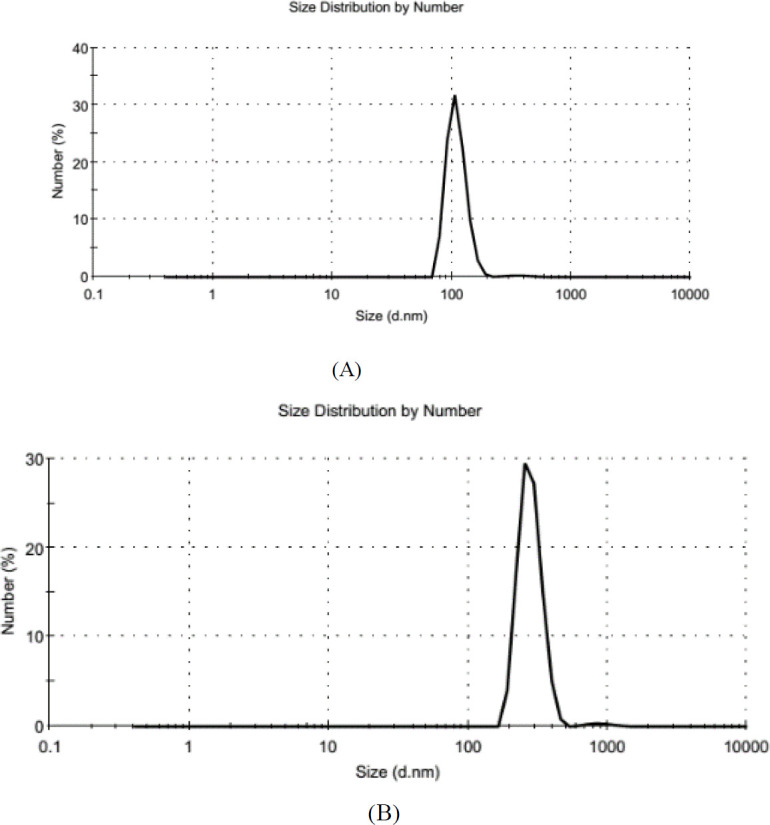
The size distribution by intensity of chitosan microparticles and Stx2B-loaded chitosan microparticles. (A) The mean size of chitosan microparticles is 215 nm, (B) The mean size of Stx2B-loaded microparticles is about 545 nm

**Figure 3:**
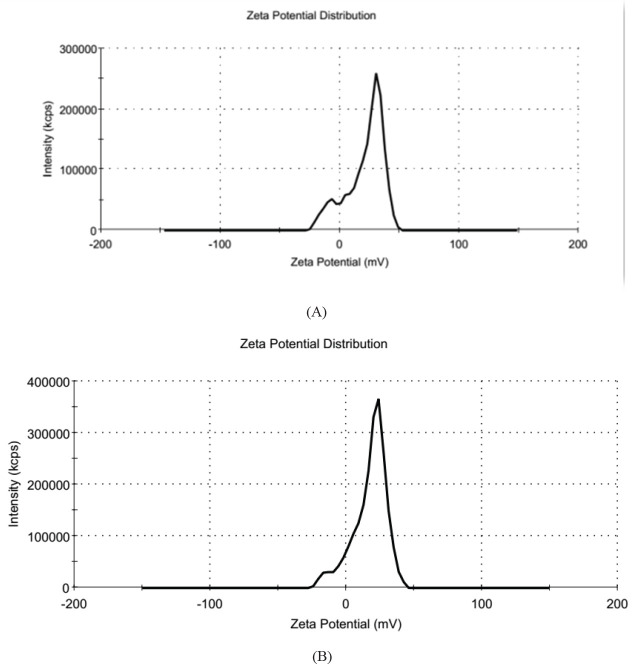
Zeta potential distribution of chitosan microparticles and Stx2B-loaded chitosan microparticles. (A) Chitosan microparticles exhibited a zeta potential with 21.1 mV, (B) Stx2B-loaded microparticles exhibited a zeta potential with 17.4 mV

The distribution and average size of prepared batches of microparticle suspension were assessed using the Zetasizer. Figure 2A represents the size distribution in a typical batch of microparticles with a mean diameter of 215 nm and narrow size distribution (polydispersity index) of 0.266. The size of Stx2B-loaded microparticles was about 545 nm, with a narrow size distribution of 0.39. (Figure 2B). Zeta potential, indicating surface charge can significantly influence particle stability in suspension through the electrostatic repulsion between particles, showed that the surfaces of chitosan microparticles have a positive charge of about 21.3 mV (Figure 3A). In comparison, that of Stx2B loaded microparticles exhibited about 17.4 mV (Figure 3B).

***In vitro release profile from Stx2B-loaded chitosan microparticles***

**Figure 4:**
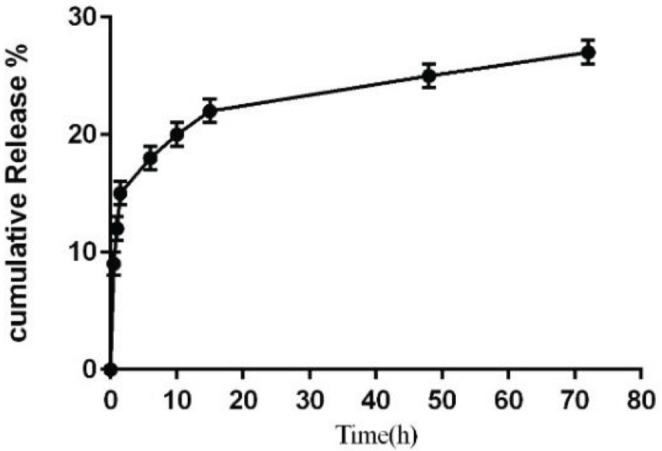
*In vitro* Stx2B release from chitosan microparticles: Stx2B-loaded chitosan microparticles crosslinked with TPP solution

Our analysis revealed that approximately 10% of the loaded Stx2B was released within 1 hr of incubation in PBS. Disassociation of loaded cargo from the delivery microparticle platform is dictated mainly by the strength of the ionic bond between them, which is affected by the ionic condition of the medium. In this study, a pH of 7.2 was chosen to mimic the bodily fluid conditions to assess the disassociation rate of Stx2B from the loaded chitosan microparticles (Figure 4). This experiment indicated three separate phases of release for each formulation tested. These phases include a rapid release of 15%, which is attributed to the drug desorbed from the particle surface, followed by a plateau phase for 15 hr representing dissociation of the drug from the nanoparticle matrix, and a final phase of continued release of the drug due to dispersion and degradation of the polymer.

***Antibody responses to immunization***

**Figure 5:**
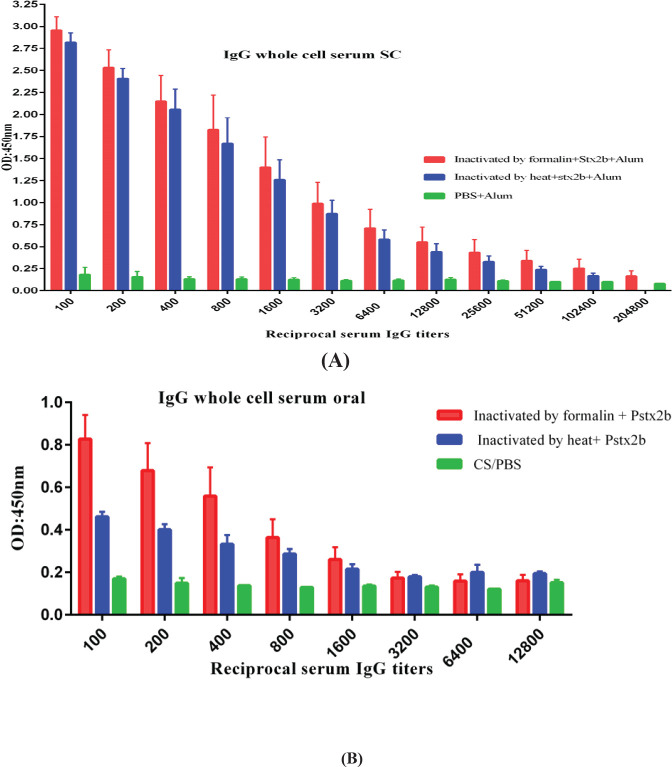
Specific serum IgG following oral vs. subcutaneous immunization with inactivated bacteria. Significant differences (*P*˂ 0.05) were obsereved in antibody titer between immunized and control mice groups. Differences in antibody titers of oral and subcutaneous groups were also significant (*P*˂ 0.05) (A) IgG titer whole cell in immunized group subcutaneously,(B) IgG titer whole cell in immunized group orally (Pstx2b means Polymerized stx2b)

**Figure 6:**
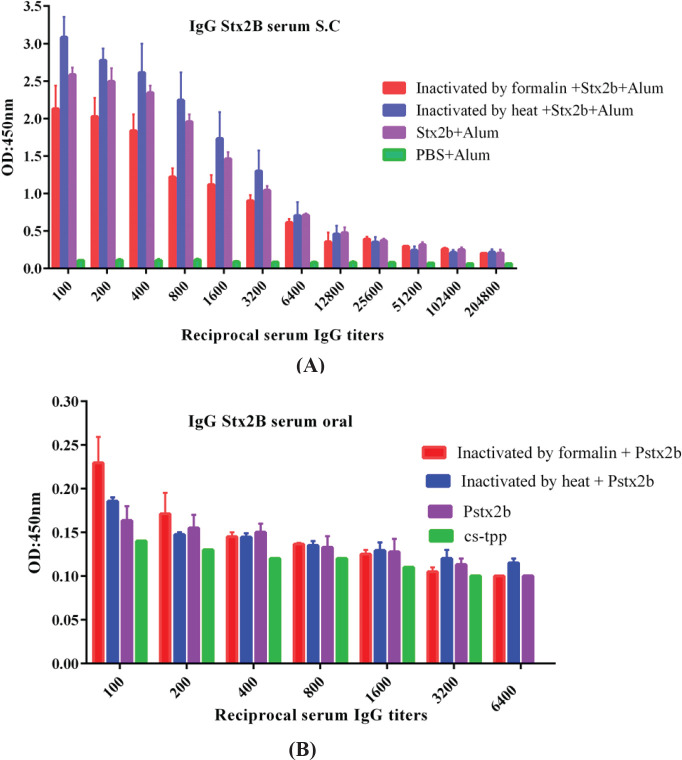
Specific serum IgG following oral vs. subcutaneous immunization with Stx2B. There were significant differences in antibody titer between immunized and control mice groups (*P*˂0.05). Differences in antibody titers of oral and subcutaneous groups were also significant (*P*˂0.05) (A) IgG titer Stx2B in immunized group subcutaneously (B) IgG titer Stx2B in immunized group orally

**Figure 7:**
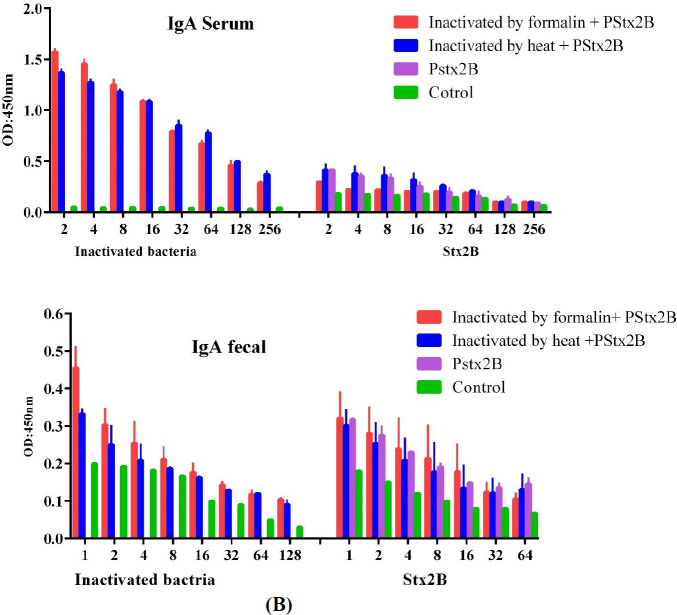
*Escherichia coli *O157:H7 and rStx2B specific serum and fecal IgA following oral immunization(A) Serum IgA titer whole cell and Serum IgA titer Stx2B in different mice groups at 7 days post-final immunization, (B) Fecal IgA titer whole cell and Fecal IgA titer Stx2B of different mice groups at 7 days post-final immunization

Significant IgG production was observed in the sera of immunized mice with either subcutaneous or oral immunizations. Compared with the control group, significantly higher and-* E. coli* O157:H7 and Stx2B IgA were observed in both sera and feces of mice immunized via oral vaccination. ELISA results are shown in Figures 5, 6, and 7.

***IgG Antibody response in serum***

Significantly higher levels of specific IgG towards *E. coli* O157:H7 and Stx2B were observed in the sera of mice immunized orally and subcutaneously versus the control group (Figures 5 and 6).

***IgA antibody responses in serum and fecal extracts***

Oral immunization resulted in significantly higher specific and- *E. coli* O157:H7, and anti-Stx2B IgA titers (*P*< 0.05) were seen after oral immunization in the fecal matter and sera of immunized mice versus the control group (Figure 7). However, this increase was lower in frequency and magnitude compared with the IgG response.

***Mice challenge with E. coli O157:H7***

**Figure 8:**
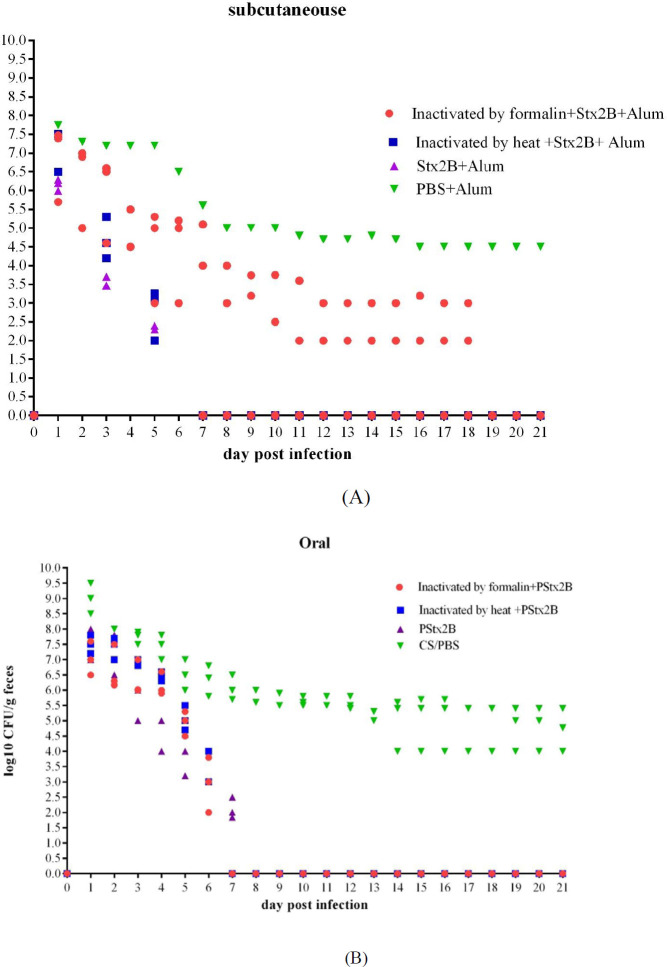
*Escherichia coli *O157:H7 shedding in feces following subcutaneous (A) and oral (B) administration in mice. Immunized and non-immunized mice were orally fed 1010 *E. coli *O157:H7 and shedding was monitored in the feces for 21 days. Differences were considered significant whenever *P*<0.05. The limit of detection for plating was 1010 CFU/0.1 g feces. Data have been repeated in separate experiments (*P*<0.05)

To assess the vaccine’s efficacy, subcutaneously and orally immunized mice were orally challenged with 10^10^ CFU of *E. coli *O157:H7. Shedding results are summarized in Figure 8. 

Results demonstrated that while immunized mice had significantly lower shedding of* E. coli *O157:H7 with gradual decline and complete stop after seven days, mice in the control group continuously shed high levels of bacteria (10^4^-10^8 ^CFU) throughout the weeks (Figure 8A,B).

Bacterial colonization showed a significant difference between immunized and non-immunized mice as assessed by Fisher’s exact test (*P*=0.050).

**Table 1:** Primer sequences used in this experiment for strain confirmation and Stx2B cloning

PCR product (length; bp)	Sequence	Primer
239	5- GTGCTTTTGATATTTTTCCGAGTAC -3	rfbE forward
239	5- TTTATATCACGAAAACGTGAAATTG -3	rfbE reverse
210	5-GATCCG**GGATCC**ATGGCCGACTGTG-3	stx2B forward
210	5-TGCTTC**AAGCTT**TTAATCATTGTTGAACTGAACTTC-3	stx2B reverse

**Table 2:** Route of administration and antigens quantitation for immunization of different mice groups

Immunization groups	Route of administration	Antigens quantitation
Inactivated *E.coli* O157:H7 by formalin plus chitosan coated Stx2B	Oral	10^9 ^bacteria + 70µg protein
Inactivated *E.coli* O157:H7 by heat plus chitosan coated Stx2B	Oral	10^9 ^bacteria + 70µg protein
chitosan coated Stx2B	Oral	70µg protein
PBS+CS/TPP	Oral	0.01M PBS
Inactivated *E. coli* O157:H7 by formalin plus Stx2B and Alum	Subcutaneous	10^8 ^bacteria + 20µg protein
Inactivated *E. coli* O157:H7 by heat plus Stx2B and Alum	Subcutaneous	10^8 ^bacteria + 20µg protein
Stx2B plus Alum	Subcutaneous	20µg protein
PBS plus Alum	Subcutaneous	0.01M PBS +Equal volume Alum

## Discussion

Although EHEC was categorized as pathogenic bacteria in the 1980s, to date, there is no effective strategy for its control. The conventional use of antibiotics is ineffective and can enhance its pathogenicity, which could be related to the worldwide outbreaks of EHEC infections (25).

*E. coli *O157:H7 is an important cause of foodborne diseases. Besides the public health concerns, economic loss due to *E. coli* O157:H7 is severe (26).

Various strategies, including vaccination and antibiotic therapy, have been used to control the widespread infections of this bacteria. However, these strategies have not been successful, and yet, there is no effective control for *E. coli* O157:H7 outbreaks (27). Thus, most therapeutic regimens aim to reduce disease severity, prevent systemic complications, and control symptoms. Based on this, developing an effective strategy to control this disease and reduce outbreaks of EHEC is vital. Given that ruminants are the main reservoir of bacteria and the leading source of infection in humans, pre-slaughter vaccination of cattle can significantly reduce the chance of infection and disease outbreaks in humans (28).

Several virulence factors are identified in *E. coli* O157:H7 and are known as the mechanism of bacterial colonization in the intestinal mucosa of humans and animals. Bacterial attachment and effacement of the surface of intestinal microvilli is the leading cause of attaching and effacing (A/E) lesions (29). Thus providing immunity against these factors and other unknown mechanisms of colonization using inactivated bacterial vaccines can be an economical and potentially safe approach to overcome these problems and prevent the spread of the disease.

In this method of vaccination, the bacteria are inactivated and delivered to the immune system to induce and stimulate an efficient and protective response (13).

An appropriate bacterial inactivation technique can significantly impact the vaccine’s success rate. Various strategies have been used; among them, formalin and heat inactivation are effective and result in a comprehensive and protective immune response (30).

Since the production of inactivated whole bacterial vaccines is cost-efficient, cattle can be vaccinated using this approach to decrease bacterial shedding and reduce the risk of *E. coli* O157:H7-related infection and disease in humans. The toxin is one of the primary pathogens of the bacteria, and it is conserved among this family. Previous studies have shown that immune response against this subunit can protect *E. coli* O157:H7 HUS (31). Moreover, since the B subunit is the primary mechanism of bacterial attachment, providing immunity against it will prevent bacterial colonization (32). Hence, here we have selected this subunit to develop our candidate vaccine. 

The most likely explanation is that the Stx2B pentamer is only marginally stable in the absence of the A subunit; thus, when used as an immunogen, it is not able to raise specific Abs against conformational epitopes that are located primarily at the interfaces between monomers of the pentamer (31). 

In addition to selecting a proper antigen for vaccine production, selecting an appropriate delivery platform also plays a major role in the vaccine’s success. Among various delivery platforms, chitosan microparticles are a successful platform for the oral delivery of vaccines. This is due to their unique properties, including preserving the cargo within its matrix, which protects against environmental and enzymatic degradation, especially in the stomach and intestine. Moreover, chitosan microparticles can attach to the mucosal surface of the intestine and maintain a presence in the gastric region to provide long-lasting interaction between the cargo antigen and the immune system, which can result in a more successful immune response. It has been shown in previous studies that antigen-presenting cells can be activated by chitosan and its derivatives to stimulate cytokine secretion and provide a balanced Th1/Th2 response (33).

This study combined inactivated bacteria with Stx2B to improve antigenicity and immune responses. Although immunization with inactivated bacteria and recombinant Stx2B is shown to provide immunity against *E. coli *O157:H7 challenge, a synergistic effect was observed with the combination of inactivated bacteria and recombinant Stx2B/chitosan coated Stx2B. Here we employed two control groups to assess the efficacy of six different vaccine formulations. We used two different delivery approaches, oral administration of whole *E. coli* O157:H7 bacteria combined with Sx2B/chitosan coated Stx2B and subcutaneous delivery of formalin or heat-inactivated bacteria. 

After live bacteria, a significant reduction in total bacterial shedding was observed with all vaccine formulation challenges (Figure 8). However, better immune response and protection were seen when the formalin inactivation technique was used in the oral delivery approach.

This could be attributed to the fact that in formalin-inactivated cells, surface antigens are provided to the immune system without any conformational change, whereas in heat-inactivated methods, surface antigens are conformationally altered.

Moreover, the immunogenic capacity of the formalin-inactivated bacteria sublimated with chitosan encapsulated Stx2B, which resulted in significant enhancement when orally delivered to mice. The challenge test’s humoral and mucosal immune responses showed that the combined vaccine provided stronger protection in mice against *E. coli* O157: H7.

Furthermore, IgG levels in combination groups were higher than in formalin or heat-inactivated groups alone.

Fecal shedding of *E. coli *O157:H7 was assessed to see the influence of inactivated bacteria and Stx2B immunization on the colonization in mice. The lowest amount of shedding was observed with the mice groups orally immunized with formalin-inactivated bacteria combined with chitosan-coated Stx2B, followed by the mice vaccinated subcutaneously using heat-inactivated bacteria along with Stx2B. Since mice in the control groups did not show bacterial shedding below 10^4^CFU per gram of fecal matter, it can be concluded that vaccination resulted in a significant reduction of bacterial colonization of *E. coli *O157:H7 in mice and has reduced mice-to-mice transmissions. However, the clearance rate may not have considerably impacted infected individuals.

## Conclusion

Our findings indicated that using inactivated bacteria along with rStx2B is a better immunization against EHEC infections than either method alone.

Since orally administered vaccines against pathogenic gut bacteria greatly reduce the burden of diarrheal diseases, therefore we recommend oral immunization with formalin-inactivated bacteria along with chitosan-coated Stx2B as a superior immunogen among all groups under our investigation.

## Authors’ Contributions

NA was the main researcher. SLM Supervised the research. JA and SN were research advisors.

## Conflicts of Interest

The authors declare no conflicts of interest.
